# One-Step Production of PEM Membranes with a Recombination Interlayer Using a Three-Layer Slot Die

**DOI:** 10.3390/membranes16100309

**Published:** 2026-09-23

**Authors:** Andrea Hintzen, Markus Stähler, Lukas Rein

**Affiliations:** Institute of Electrochemical Process Engineering: IET-4, Institute of Energy Technologies: IET, Forschungszentrum Jülich GmbH, 52425 Juelich, Germany; m.staehler@fz-juelich.de (M.S.); l.rein@fz-juelich.de (L.R.)

**Keywords:** PEM water electrolysis, membrane electrode assembly (MEA), recombination catalyst, gas crossover, hydrogen permeation, three-layer slot die coating, roll-to-roll manufacturing

## Abstract

Using membranes with recombination catalyst layers in PEM water electrolyzers effectively reduces the formation of explosive gas mixtures. Slot die coating is an established method for producing such functional layer systems. This work demonstrates the fabrication of a recombination catalyst-containing membrane in a single step using a three-layer slot die. The membrane is made of an insulating ionomer layer on the top and bottom layer and an intermediate layer containing a recombination catalyst. Cell experiments confirm that the membrane effectively reduces hazardous hydrogen concentrations in the anode circuit of the PEM electrolysis cell.

## 1. Introduction

Polymer electrolyte membrane (PEM) water electrolysis (WE) is considered a promising technology for sustainable hydrogen production [1,2,3,4]. The central component of a PEM WE system is the membrane electrode assembly (MEA). In terms of scalability, throughput, coating quality and industrial transferability, roll-to-roll (R2R) processes in combination with continuous coating processes, such as doctor blade, rod or slot die coating, have proven to be particularly efficient for MEA mass production [5,6]. Current challenges in these R2R production processes include, among other things, further optimizing the processes for manufacturing high-performance MEAs [7].

Numerous studies have focused on developing and optimizing MEAs. One important way of increasing efficiency is using thinner membranes to reduce ohmic resistance and enable higher current densities [8,9,10]. However, reducing the membrane thickness increases hydrogen permeation to the anode, which can lead to the formation of potentially explosive gas mixtures [9,11].

Integrating recombination catalysts (RC) into the membrane has proven to be an effective mitigation strategy. For example, platinum-containing catalysts inside the membrane enable the catalytic conversion of permeated hydrogen and oxygen to water, thus contributing to increased safety. Several approaches have been reported for incorporating the catalyst into the membrane [12,13,14,15,16,17]. Klose et al. applied an ionomer/Pt dispersion to a commercial membrane via spray coating, then laminated it with a second membrane [13]. Martin et al. demonstrated that the positioning of the RC has a particularly significant influence on the reduction in the hydrogen content in the oxygen stream in fully spray-coated membranes [18]. Mirshekari et al. introduced a recombination layer between two commercial membranes using reactive spray deposition technology, investigating the performance and durability of a two-cell PEM WE stack [17]. Another approach involves incorporating Pt into the membrane, as demonstrated by Garbe et al. [15,16].

With the goal of manufacturing high-performance MEAs for PEM fuel cells using R2R processes, the question arises as to which approaches can be applied. As we were able to demonstrate in an earlier study, slot die coating processes can be used to integrate the RC into the membrane in a scalable R2R process [14]. This involved alternately depositing an insulating layer and an RC-containing layer onto the anode, followed by an assembly step involving a membrane and a cathode. While this method worked well in an R2R process, it requires multiple coating and drying steps, which makes the entire manufacturing process complex and time-consuming [14]. The method does not yet meet the requirements for an optimized and simplified process flow [7]. Consequently, the alternating application of the different membrane phases must be further simplified so that as many layers as possible can undergo a single drying step.

Multi-layer slot die coating processes, which are already used in lithium-ion battery development to build multi-layer systems in a single step [19,20,21,22], offer an attractive path toward further simplifying the MEA manufacturing process chain and even eliminating the need for a separate membrane.

Against this background, the present study aims to simplify the scalable production of thin, functional membranes for high-performance electrolysis using simultaneous multi-layer coating. Figure 1 illustrates this idea and provides a comparison of the manufacturing effort for an RC-containing membrane produced using a single-layer slot die—requiring three coating and drying steps—versus the effort involved when using a three-layer slot die. This approach eliminates two complex coating and drying steps, representing a significant increase in process efficiency. The use of multi-layer slot dies is therefore the logical next step toward the simplified R2R production of functionalized multi-layer membranes for the PEM WE.

In the present study, a three-layer membrane (3LM) was produced in a pilot plant with a 200 mm wide three-layer slot die. The die’s three separate slots were loaded with pure Nafion™ dispersion, Nafion™ dispersion with RC, and pure Nafion™ dispersion, respectively. After drying and annealing, the membrane was processed into an MEA and analyzed electrochemically in a PEM electrolysis cell. Compared to an MEA with a single-layer membrane (1LM) without a RC, only a negligible amount of permeating hydrogen was observed, despite the membrane’s thickness of only about 19 µm.

## 2. Materials and Methods

Membrane Manufacturing—The membranes were produced in a pilot plant (JHT—Jakobs-Houben Technologie GmbH, Geilenkirchen, Germany), which can be operated either as an R2R coating machine or with a conveyor belt for the sequential coating and drying of multiple layers on a reusable decal sheet [23].

A 200 mm wide single-layer slot die (TSE Troller AG, Murgenthal, Switzerland) was used for the fabrication of the 1LM. The fabrication of the membrane onto fabric-reinforced polytetrafluoroethylene (PTFE) (CF206, Saint-Gobain, Kilrush, Ireland) was performed using a method similar to that described by M. Stähler et al. [24]. The process was scaled up to a width of 200 mm using the parameters summarized in Figure 2 and Table 1. The 1LM was prepared by applying a Nafion™ ionomer dispersion (D2021, Chemours, Wilmington, DE, USA) diluted to 19 wt.% with a 1:1 mixture of 1-propanol and deionized water.

For the single-step 3LM coating, a three-layer slot die (TSE Troller AG) with a width of 200 mm was installed in the R2R pilot plant. Each slot of the die was filled with a dispersion via a separate connection, which was metered into the gap by a syringe pump (LEGATO^®^ 210, KD Scientific, Hollisten, MA, USA). Layers 1 and 3 were produced using the same dispersion as in the 1LM. The dispersion containing the RC that formed layer 2 was prepared as described in our previous study [14]. The gap and slot die settings for the 3LM were adjusted as shown in Figure 2 and Table 1. Subsequent drying was performed at approximately 80 °C.

Each membrane was annealed using a PTFE calendering roll inside the plant. For this purpose, the roller pair was heated to 150 °C, and the membrane was passed through at a minimum line speed of 0.1 m/min at a pressure of 2 bar and a gap of 160 µm. Finally, the membranes were delaminated from the substrate and wound onto a protective layer. For the MEA processing, a 90 × 90 mm^2^ piece was used in each case.

MEA Preparation—The MEAs were fabricated with in-house-produced anodes and cathodes and processed via the decal method. The anode loading was (0.95 ± 0.05) mgIr cm^−2^ (IrO2, Premion, Alfa Aesar, Haverhill, MA, USA) and the cathode loading was (0.30 ± 0.05) mgPt cm^−2^ (60% Pt on Ketjenblack EC-300J, Fuel Cell Store, TX, USA), with an electrode area of 4.2 × 4.2 cm^2^ for each. This preparation method was described for the same materials in detail in our previous work [14,25]. For clarity, the sample produced using the single-layer slot die is designated “1LM,” and the MEA produced using the triple-layer slot die is referred to as “3LM.”

H_2_-in-O_2_ Measurement—For safety reasons, the test rig for the electrochemical characterization needed to be configured to trigger a shutdown if the H_2_ volume fraction in air or O_2_ exceeded 50% of the LEL (lower explosive limit). In order to enable measurements of H_2_ volume fractions higher than 2 vol-% in the anode oxygen stream, it was necessary to dilute the anode gas with an O_2_ flow rate V_S_(O_2_) of 200 mL_n_/min (O_2_, ultrapure, Linde) directly at the cell outlet. This ensured that the safety conditions were met. The H_2_-in-O_2_ volume fraction c was then measured in the diluted oxygen stream using a thermal conductivity detector (FTC300, Messkonzept GmbH, Frankfurt am Main, Germany, measurement range 0–3 vol-%, accuracy after calibration is: 0.02 × (read value) + 0.0001). Prior to the gas entering the sensor, the water vapor present was condensed out, ensuring the gas dew point remained constant at (−1 ± 0.5) °C and the water vapor content within the thermal conductivity detector stayed constant across all measurements.

The thermal conductivity detector was calibrated using calibration gas (H_2_-in-O_2_ volume fraction, relative accuracy ±2%, Linde) adjusted to the same dew point. The H_2_-in-O_2_ volume fraction at cell output c_cell_ was calculated from the measured values c and the uncertainty was determined via error propagation.

(1) c_cell_ = (V_s_(O_2_) + I × k)/(V_s_(O_2_) + I × k/c)

with

(2) k = T_s_ × R/(4 × F × p_s_)

I: Electric current

T_s_: Standard temperature (273.15 K)

p_s_: Standard pressure (101,325 Pa)

F: Faraday constant

R: Universal gas constant

All details regarding the setup and calculations are provided in [25,26]. The measurement uncertainties for the H_2_ volume fractions reported in this manuscript correspond to a single standard deviation.

Calculation of the permeation current density j_P_(H_2_)—Using the measured current density j, Faraday’s constant F, and the active area A, the electrochemically generated O_2_ can be calculated via Faraday’s law:

(3) n′(O_2_) = j × A/(4 × F)

The permeated hydrogen flow n′_P_(H_2_) can be calculated using this oxygen flow and the measured H_2_-in-O_2_ volume fraction c_cell_:

(4) n′_P_(H_2_) = c_cell_ × n′(O_2_)/(1 − c_cell_)

The permeation current density j_P_(H_2_) is then calculated by

(5) j_P_(H_2_) = 2 × F × n′_P_(H_2_)/A

Electrochemical Characterization—The permeation of H_2_ to the anode is of particular interest in determining the reliability of the RC when the membrane is fabricated in a single step. To this end, both types of MEAs were characterized using the same in-house-developed test cell setup and measurement procedures [25,27]. To ensure uniform contact pressure distribution across the entire cell surface, an additional 1 mm-thick iridium-sputtered titanium (Ti)-PTL was added to a 350 µm thick one (Bekipor^®^ Titanium, 2GDL-40-1.0 and 2GDL-10-0.35, Bekaert, Zwevegem, Belgium) on the anode, and on the cathode side, a C-PTL with MPL (H15C14, Freudenberg Group, Weinheim, Germany) and a 1 mm thick, platinum-sputtered Ti-PTL (2GDL-40-1.0) were used [25]. Five polarization curves were measured sequentially at 80 °C. The applied cell voltage started at 1.45 V and was increased to 1.75 V in 0.05 V increments every 15 min. To capture hysteresis effects, the cell voltage was subsequently decreased in 0.1 V increments every 15 min. Preliminary tests had shown that the steady state regarding current density and H_2_ volume fraction is reached after 10 min. The reported measured values represent the averages of the last 5 min. The polarization measurements were accompanied by beginning-of-test and end-of-test impedance measurements at 1.45 V to determine the high frequency resistance (R_HF_) value. The measurements were taken with a workstation Zennium Pro from Zahner-Elektrik GmbH & Co. KG (Kronach, Germany), which had already been used in our previous work [25].

## 3. Results and Discussion

### 3.1. Membrane Manufacturing

Based on prior experience, the 1LM coating settings were developed on a TableCoater (TSE Troller AG, Murgenthal, Switzerland) and scaled up from laboratory experiments to the R2R plant. With the previously specified parameters for coating, drying, and annealing, the coating machine produced a membrane that was approximately 19 µm thick, 2.5 m long, and 200 mm wide. After passing through the calendering unit, the 1LM was delaminated from the substrate and wound onto a protective layer (see Figure 3c).

The development and initial production of the 3LM membrane was carried out directly in the R2R plant; since the three layers must be dosed one after the other at a certain distance (see Figure 3a), the development was not possible using a TableCoater at laboratory scale.

For the 3LM production, first, the dosing of the bottom layer was started, followed by the next pump at 100 mm intervals, building the three layers one by one as films (see Figure 3a,b). Initial attempts without offset ΔG_L2_ and ΔG_L3_ (see Figure 2) resulted in complete mixing of the three films. By adjusting these values, stable films were produced. In this way, a 3 m × 200 mm membrane with a thickness of approximately 19 µm was manufactured; however, this showed a slightly uneven distribution of catalyst particles across the width of the coating. This inhomogeneity, which arose during the drying process, can be seen in Figure 3d, where the membrane was peeled off the substrate and wound up.

In the initial attempts, the functionality of the membrane was prioritized. The goal was to demonstrate whether this method could be an improvement over individually applied layers. Therefore, the membrane was processed into MEAs for electrochemical characterization.

### 3.2. Electrochemical Characterization

As in our previous work, in which the recombination layer was incorporated into the membrane using a more complex method, electrochemical characterization of the samples was performed, focusing on the polarization curves, IR-corrected polarization voltages, and H_2_-permeation analysis, as summarized in Figure 4a–d [14].

Figure 4a shows the results of the first and fifth polarization measurement cycles of the 1LM and 3LM samples. As is known from other studies, an increase in performance is evident over the course of the measurements, which is comparable for both membrane types. The 1LM shows slightly better performance than the 3LM. Both lie at around 4.8 A/cm^2^ and 4.5 A/cm^2^, respectively, at 1.75 V, comparable to the results produced with commercially available 20–25 µm thick membranes [14].

When interpreting the measured R_HF_ values, it should be noted that, due to the very thin membrane, the values are very low, at approximately 30 mΩ cm^2^ and 35 mΩ cm^2^, respectively. Given the measurement uncertainty of 3.2 mΩ cm^2^ (standard deviation, see Reference [14]), it is very difficult to assess the differences between the polarization curves.

The corrected polarization voltages and their uncertainties, which were calculated using error propagation, are shown as error bars in Figure 4b, and were obtained from the following equations:

(6) U(iR_HF_-free) = U_c_ − R_HF_ × j

(7) dU(iR_HF_-free) = [(dU_c_)^2^ + (dR_HF_ × j)^2^ + (R_HF_ × dj)^2^]^0.5^

with dU_c_ = 0.5 mV, dR_HF_ = 3.2 mΩ cm^2^, and dj = 1% of the measured value.

The results indicate that the corrected polarization values obtained cannot be distinguished from one another within the uncertainty limits, but are consistent with previous results within those limits [14].

The data presented in Figure 4c indicate that the effect of the implemented recombination layer on the H_2_ volume fraction in the oxygen-path is also consistent with previous work. The H_2_ permeation of the 1LM sample results in a volume content of more than 3 vol-% in the range of small current densities (j < 0.2 A cm^−2^) in both the first and fifth cycles. Considering that the lower explosion limit of H_2_-in-O_2_ is 4%, and that some technical facilities only allow 50% of this value for safety reasons, this MEA could not be used without further measures. Even at higher current densities, the amount of permeating hydrogen increases over the course of the cycle. While the volume fraction of H_2_ in O_2_ is still at 0.5 vol-% at the beginning, it doubles to 1 vol-% over time, and based on previous observations, it can be assumed that the hydrogen permeation will continue to increase [14,25,28].

Examination of H_2_ permeation in the 3LM-MEA shows only a negligible amount of H_2_ in the anode circuit. At low current densities, the volume fraction is 0 vol-%, taking the measurement uncertainties into account. At higher current densities, it is less than 0.15 vol-%. Thus, the 3LM-MEA behaves as expected.

By applying Equations (1)–(4), the H_2_-volume fraction data c_cell_ can be used to calculate the permeation current density j_P_(H_2_) for the 1LM and 3LM samples, presented in Figure 4d. While the blue curve (3LM) again demonstrates the functionality of the RC, the red line gives an impression of the Faraday losses (j_P_(H_2_)/j) during operation because of the H_2_ permeation. Up to current densities of 4.8 A/cm^2^ (U_cell_ = 1.75 V), the permeation current density is smaller than 20 mA/cm^2^, and therefore the Faraday losses are smaller than 0.5%.

The results of the presented study show that 19 µm thin membranes containing a recombination catalyst for PEM WE can be produced in a single R2R manufacturing step. This enables current densities of up to 4.5 A cm^−2^ at 1.75 V and Faraday losses of less than 0.5%.

## 4. Conclusions

Previous work on membranes produced by using slot die coating processes and the scalable production of MEAs containing a RC layer has demonstrated a process chain that enables the production of thin MEAs that can be operated safely. Therefore, using a three-layer slot die to simplify and reduce the number of process steps was a reasonable step to take to produce MEAs more efficiently.

Therefore, the present study shows the production of a RC-containing membrane that was manufactured in one step with the help of a three-layer slot die. In this way, the membrane was also manufactured in a scalable R2R process. Electrochemical investigations revealed that the reduction in permeating hydrogen is just as effective across the entire current density range as the previously used method [14]. From a process engineering perspective, the triple-layer slot die coating process is therefore significantly more sophisticated than the process presented previously. Four process steps—two coating and drying cycles—are eliminated, as the coating and drying steps need to be performed only once instead of three times in succession. Consequently, the process should be further developed as it represents a promising approach to producing thin, high-performance MEAs that can be operated safely.

Furthermore, the use of multi-layer slot dies in the development of PEM electrolyzers and PEM fuel cells opens up exciting possibilities to produce membranes with additional functional layers that enhance the properties of the MEAs. Interesting approaches in this regard include, for example, functional layers containing cerium-zirconium oxides to extend the durability of membrane materials, as demonstrated, for instance, by the work of Zhang et al., who incorporated these layers—as well as RC interlayers—into the membrane via spray coating [29]. This process thus offers a wide range of possibilities for manufacturing high-performance MEAs using efficient R2R manufacturing processes.

## Figures and Tables

**Figure 1:**
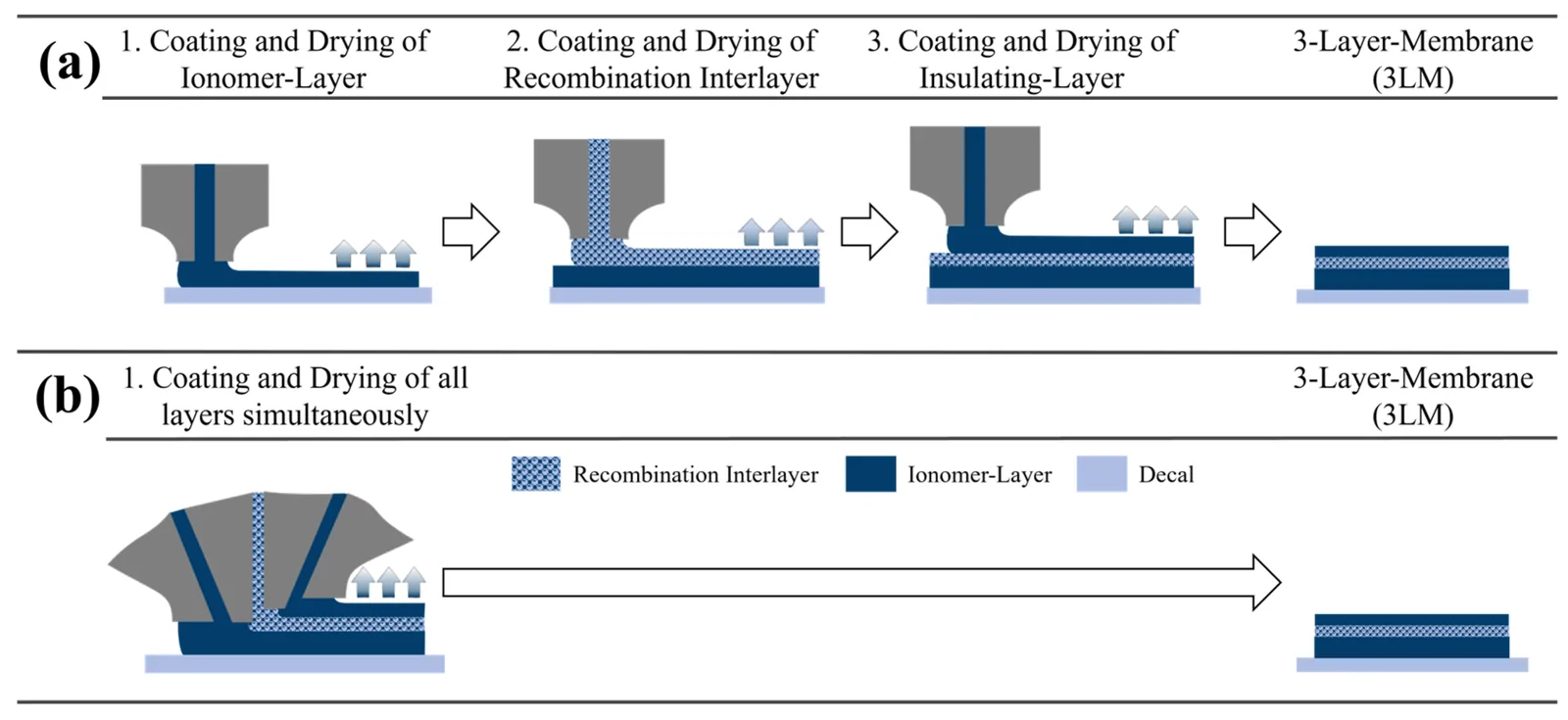
Schematic representation of the manufacturing process of a three-layer membrane (3LM) containing a RC interlayer: (**a**) using a single-layer slot die and (**b**) using the advanced method of a three-layer slot die.

**Figure 2:**
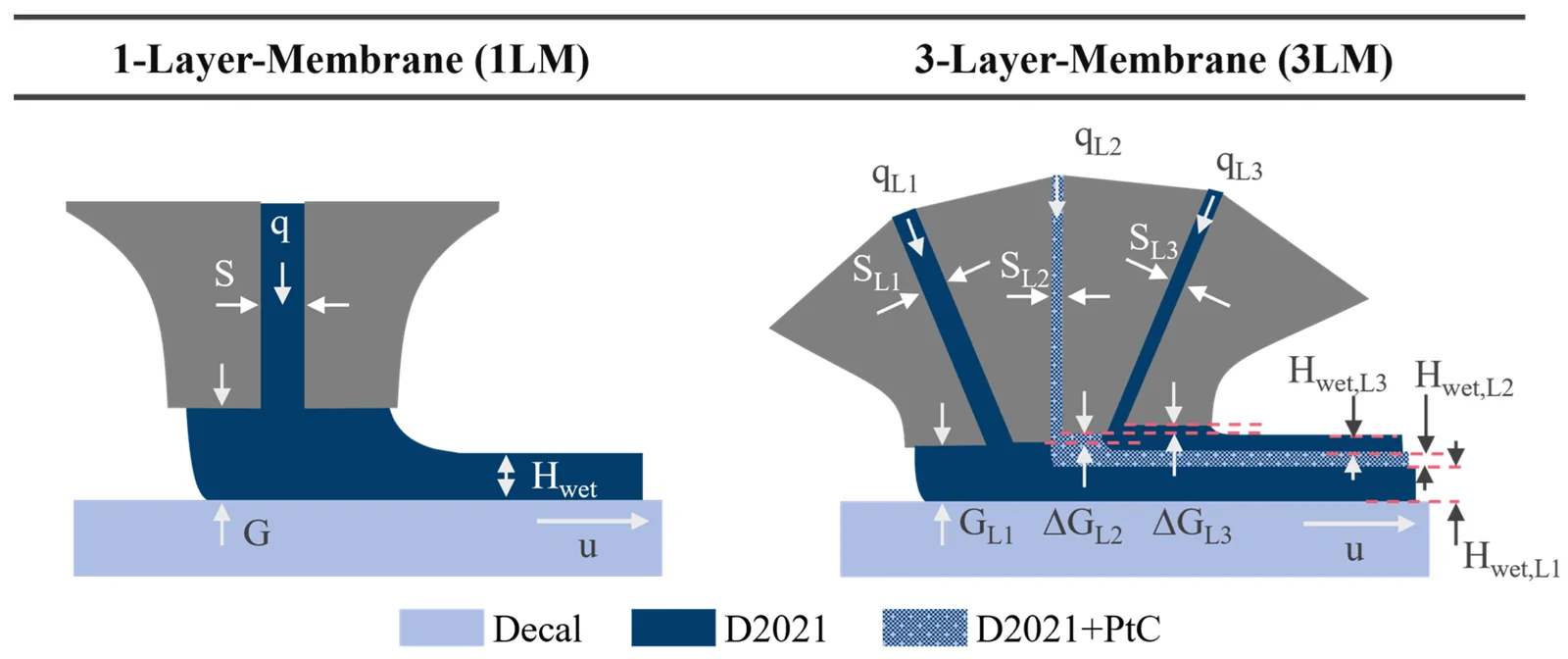
Slot die settings and coating parameters for the production of the 1LM and 3LM coatings.

**Figure 3:**
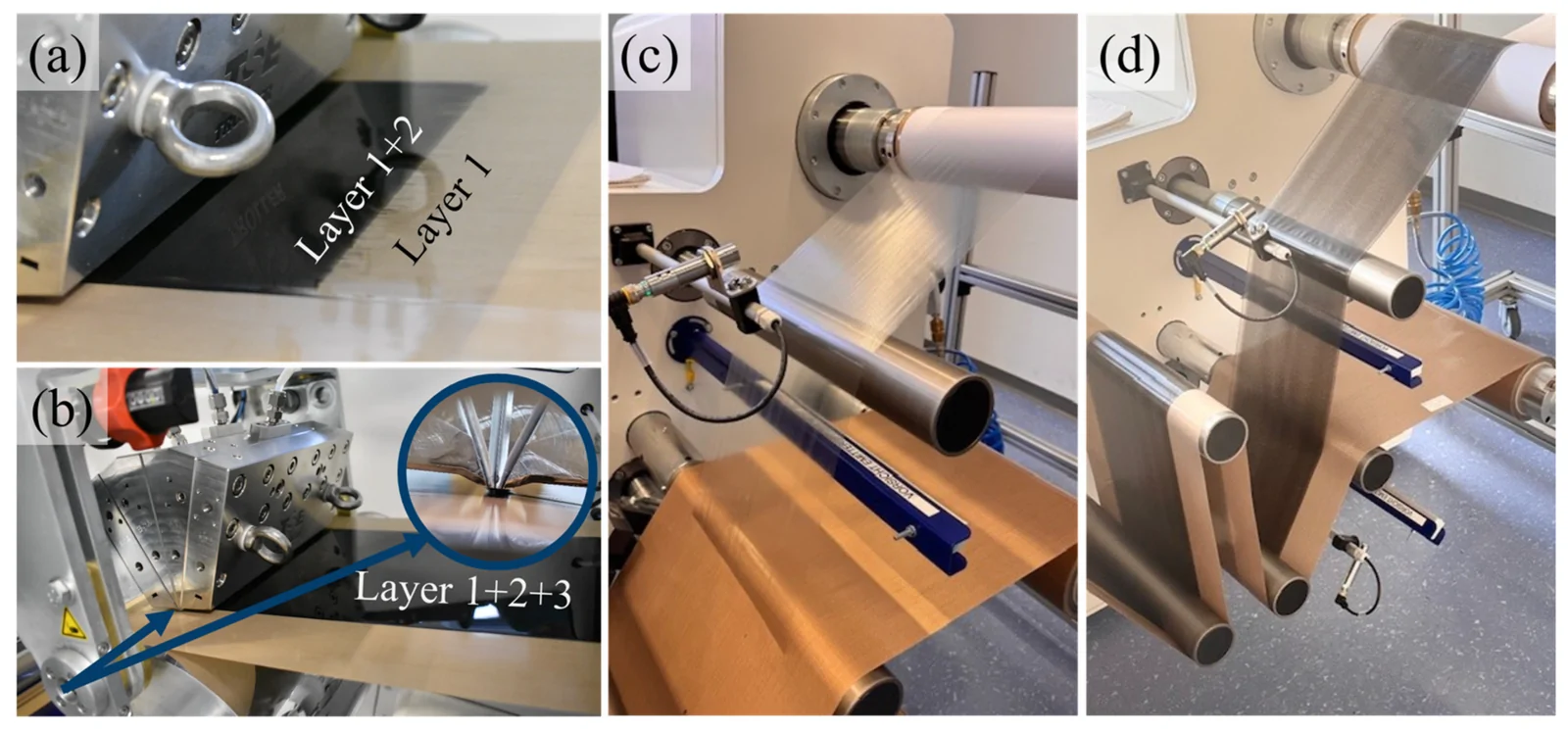
Membrane production in pilot plant scale. (**a**) Beginning of the coating process with the 3-layer-slot die. Transition from the first transparent ionomer layer to the start of the second layer containing the RC. (**b**) Three-layer coating of the membrane and the additional side view of the slot. (**c**) Delamination of 200 mm wide reference membrane with a thickness of 19 µm. (**d**) Delamination and winding of a 19 µm thick and 200 mm wide membrane coated with the three-layer slot die.

**Figure 4:**
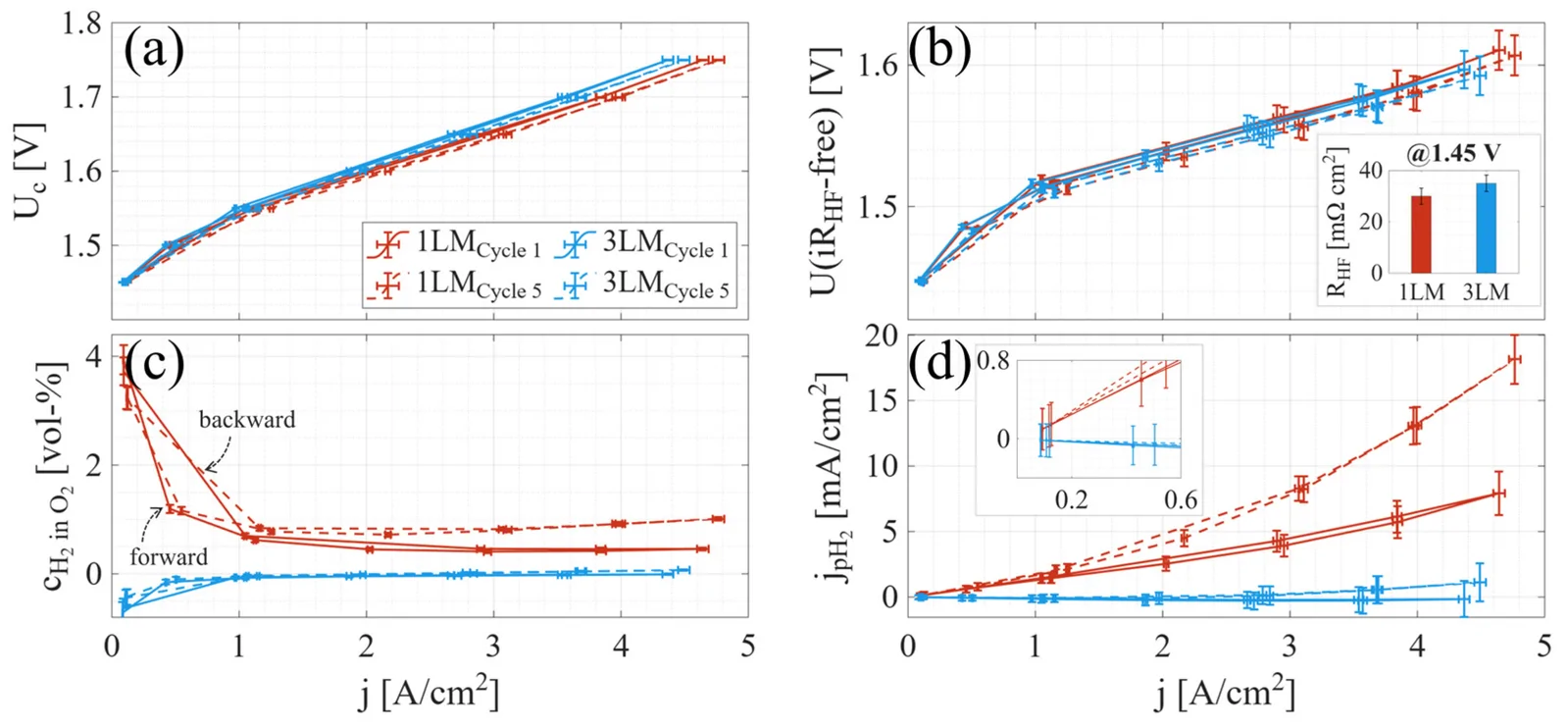
Polarization curves and hydrogen permeation data of the 1LM and 3LM samples, measured at 80 °C and ambient pressure. (**a**) Polarization curves: The solid lines represent the results of the first measurement cycle, while the dashed lines show the 5th measurement cycle. Red colors represent the data from the 1LM sample, while blue indicates the results of the 3LM sample. The cell voltage was increased in 0.05 V increments from 1.45 V to 1.75 V (forward) and then reduced back to 1.45 V in 0.1 V increments (backward). (**b**) iR_HF_-corrected polarization curves. The inserted bar chart displays the R_HF_ values for the 1LM and 3LM. (**c**) H_2_-in-O_2_ volume fractions measured in parallel. The solid lines show the results of the first measurement cycle, and the dashed lines show the results of the 5th measurement cycle. Red colors represent the data from the 1LM sample, and blue colors show the results of the 3LM sample. The small sensor drifts to negative values for current densities smaller than 0.5 A/cm^2^ are related to measurement uncertainties because of the small amount of oxygen which comes from the cell output in relation to the dilution flow of 200 mL_n_/min oxygen. Within two times the calculated standard deviation, all H_2_-in-O_2_ volume fraction values for 3LM are negligible. (**d**) Hydrogen permeation current density as a function of current density. The solid lines represent the first measurement cycle, while the dashed lines show the results of the fifth cycle. Red: 1LM sample; blue: 3LM sample. The error bars represent the single standard deviation, calculated via error propagation.

**Table 1:** Slot die settings and coating parameters for the single-layer and triple-layer coating. Coating speed u = 0.5 m/min; coating width = 200 mm.

Settings	1LM	L1	L2	L3
q [mL min^−1^]	15.8	10.6	2.6	2.6
S [µm]	200	165	100	100
G or ΔG [µm]	400	350	38	38
H_wet_ [µm]	~155	~105	~25	~25

## Data Availability

The data presented in this study are available on request from the corresponding author, but they are not publicly available.

## Abbreviations

The following abbreviations are used in this manuscript:

PEM	Polymer Electrolyte Membrane
WE	Water Electrolysis
MEA	Membrane Electrode Assembly
R2R	Roll-to-Roll
RC	Recombination Catalyst
1LM	One-Layer Membrane
3LM	Three-Layer Membrane
PTFE	Polytetrafluoroethylene
